# Robust Unsupervised Analysis of Longitudinal Functional Trajectories in Older Inpatients Reveals Stable Recovery Profiles: The AIRCOT Study

**DOI:** 10.3390/ejihpe16080111

**Published:** 2026-07-30

**Authors:** Sergio Martinez-Zujeros, Pedro J. Zufiria, Aránzazu Vázquez Sasot, María Luisa Delgado-Losada

**Affiliations:** 1 Department of Geriatrics, Hospital Universitario Cruz Roja San José and Santa Adela, 28003 Madrid, Spain; sermar01@ucm.es; 2 Ageing, Disability and Society Research Group in Ageing, Disability and Society (ENDISSCO), Complutense University of Madrid (UCM), 28223 Madrid, Spain; 3 Department of Experimental Psychology, Faculty of Psychology, Complutense University of Madrid (UCM), 28223 Madrid, Spain; 4 Departamento de Matemática Aplicada a las TIC, Information Processing and Telecommunications Center (IPTC), ETSI Telecomunicación, Universidad Politécnica de Madrid (UPM), 28040 Madrid, Spain; pedro.zufiria@upm.es; 5 Department of Rehabilitation, Hospital Universitario Cruz Roja San José and Santa Adela, 28003 Madrid, Spain; avazquezs@salud.madrid.org

**Keywords:** geriatric assessment, machine learning, cluster analysis, recovery of function, occupational therapy, rehabilitation

## Abstract

Functional decline in older adults represents a major challenge in geriatric rehabilitation, and machine learning (ML) clustering techniques may help identify functional recovery profiles and support personalized rehabilitation strategies. This study characterizes functional recovery profiles in older adults admitted for rehabilitation using unsupervised clustering techniques. A retrospective longitudinal study was conducted including 957 older adults admitted to a geriatric rehabilitation unit between 2019 and 2025. Clinical and functional variables, including the Modified Barthel Index (MBI), Daniels and Worthingham’s Muscle Testing, and Functional Ambulation Category, were collected from medical records. Considering the baseline functional status and the temporal changes in MBI scores, a clustering of the functional trajectories has been performed using a k-means algorithm based on the silhouette score. The robustness of the resulting segmentation has been evaluated by comparing alternative partitions obtained from bootstrap-sampling, hierarchical clustering, and the centroids of Gaussian Mixture Models. Four distinct functional recovery profiles were identified, showing different trajectories of independence, ambulation, and muscle strength during rehabilitation. Two clusters demonstrated favorable recovery and higher functional resilience, whereas the remaining profiles were characterized by chronic impairment or severe damage with limited recovery. These findings support the usefulness of unsupervised ML clustering techniques for identifying clinically meaningful recovery profiles and may facilitate patient stratification and individualized intervention planning in geriatric rehabilitation.

## 1. Introduction

Despite advances in early rehabilitation protocols for functional decline in older adults over the last decade, this population remains particularly vulnerable to clinical complications and loss of autonomy following acute conditions and hospitalization (Heo et al., 2019). The progressive aging of the population is exacerbating this problem, generating an increasing healthcare, economic, and social burden on health systems and long-term care services (Jones & Dolsten, 2024; Kallestrup-Lamb et al., 2024).

In recent years, artificial intelligence (AI) has provided new tools capable of optimizing clinical assessment, planning, and intervention through the analysis of large volumes of data (Bajwa et al., 2021), including the field of functional recovery in geriatrics. In this context, machine learning (ML) has emerged as one of the most relevant collection of procedures enabling the development of predictive models capable of anticipating clinical outcomes (Badawy et al., 2023), as well as stratification techniques aimed at identifying subgroups of patients with similar characteristics and clinical needs (Shin et al., 2023).

Among unsupervised learning techniques, clustering has gained increasing relevance due to its usefulness in identifying distinct clinical profiles, analyzing recovery trajectories, and improving patient stratification (Aljohani, 2024), promoting more personalized models of care. Furthermore, these techniques contribute to a better understanding of population heterogeneity and may facilitate both the interpretation of predictive models and the optimization of clinical decision-making (Elbattah & Molloy, 2017).

Despite these potential benefits, the application of these techniques in geriatric rehabilitation remains limited and continues to face important methodological challenges (Lanotte et al., 2023). The main challenge lies in the clinical heterogeneity and the coexistence of multiple etiologies underlying functional decline, including stroke, fractures, falls, and prolonged immobilization. Nevertheless, the literature addressing populations with these characteristics remains extremely limited, as most previous studies have focused on homogeneous samples involving a single cause of functional impairment (Albu et al., 2024; Kanai et al., 2026; G. Kim et al., 2025; H. Kim et al., 2022; Pérez et al., 2016; Shin et al., 2023).

However, the identification of rehabilitation profiles in geriatrics has considerable clinical relevance. If healthcare professionals were able to anticipate the functional recovery trajectories associated with a patient’s specific group, they could more easily individualize treatment, optimize available resources, and establish more realistic expectations (Stinear, 2017). Most authors in this field agree on the need to stratify the heterogeneous geriatric rehabilitation population and phenotype homogeneous patient subgroups, thereby facilitating the development of more individualized intervention protocols (Armstrong et al., 2012; Elbattah & Molloy, 2017; Kanai et al., 2026; Shin et al., 2023). Particularly noteworthy is the study by Mahmoud and colleagues, which combined clustering techniques with ML-based predictive models of functional recovery, thereby improving both predictive performance and model interpretability (Elbattah & Molloy, 2017).

Unlike other disciplines primarily focused on biological or structural recovery, Occupational Therapy (OT) uses performance in activities of daily living (ADL) as its main indicator of rehabilitation success. Since the clustering model presented here is based on the Modified Barthel Index (MBI), OT is particularly well positioned to interpret these recovery trajectories, translating data-driven profiles into therapeutic goals that directly influence patient autonomy and reintegration into their living environment (Boop et al., 2020).

This study is part of the AIRCOT project (Artificial Intelligence Red Cross Occupational Therapy), a research initiative developed at Hospital Universitario Cruz Roja in Madrid aimed at integrating AI-based methodologies into the field of OT. The project seeks to support personalized prediction of functional outcomes and optimize rehabilitation planning across different clinical conditions. By identifying distinct functional recovery profiles, healthcare professionals may provide more tailored guidance to families and design interventions adapted to the characteristics and risk level of each patient.

The innovative nature of this study lies in the use of a large cohort (n = 957) and the structured integration of clinical, sociodemographic, and laboratory variables to develop clustering techniques aimed at stratifying patients into homogeneous subgroups, thereby transforming this information into clinically meaningful information for personalized rehabilitation planning.

The main objective of this study is to characterize clinical and functional profiles of older adults admitted for functional rehabilitation using clustering techniques, to promote more individualized rehabilitation strategies.

## 2. Materials and Methods

This study followed a retrospective, observational, and longitudinal design. The protocol was approved by the Research Ethics Committee of the Complutense University of Madrid, Spain (reference number: CE_20220616-13_SAL; approval date: 13 October 2022), and all procedures were conducted in accordance with the ethical principles established in the Declaration of Helsinki and its subsequent amendments.

The sample included 957 adult patients hospitalized due to functional impairment associated with neurological or musculoskeletal conditions, as well as functional decline secondary to prolonged immobilization from different causes, who were considered suitable candidates for rehabilitation treatment based on their potential for functional recovery (Baztán Cortés et al., 2020).

Patients were enrolled at the Functional Recovery Unit of the Geriatrics Department of Hospital Universitario Cruz Roja “San José y Santa Adela” (Madrid, Spain). Clinical data were obtained between March 2019 and March 2025 and anonymized before processing and analysis. Eligibility criteria included being older than 60 years, presenting functional deterioration with a duration shorter than one month and obtaining a score less than or equal to three on the Cruz Roja Mental Scale (CRM). Patients were excluded if they required transfer to the Acute Care Unit during the first 72 h of hospitalization due to clinical instability, were unable to communicate in Spanish, or scored above 18 on the Socio-Family Assessment Scale (TSO). A TSO score > 18 constitutes one of the routine admission exclusion criteria of our Functional Recovery Unit, as these patients generally require alternative care pathways and social support resources rather than specialized inpatient geriatric rehabilitation.

**Figure 1:**
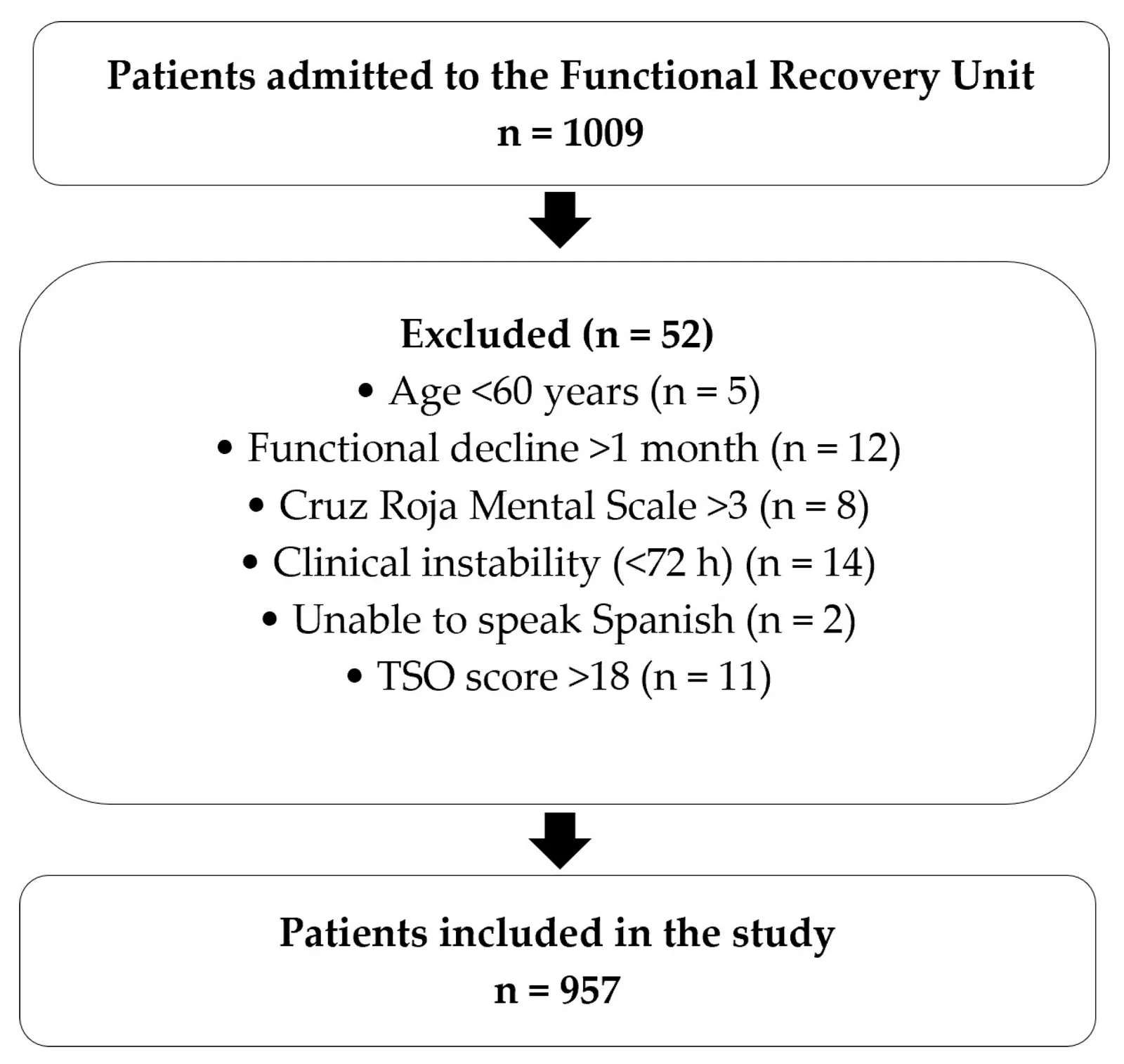
Flow diagram of participant selection.

These eligibility criteria were designed to identify older adults with sufficient rehabilitation potential while ensuring a clinically homogeneous population for the analysis of functional recovery trajectories. A detailed flow diagram describing patient screening, exclusions, data quality assessment, and final inclusion has been incorporated as Figure 1.

Patients underwent a comprehensive rehabilitation program that integrated conventional therapeutic approaches with advanced rehabilitation technologies. Standard interventions included occupational therapy, physiotherapy, speech and language therapy, and neuropsychological treatment, all coordinated within a multidisciplinary rehabilitation framework. Rehabilitation sessions were provided daily and individually adapted according to each patient’s clinical condition, functional deficits, and therapeutic goals to optimize functional recovery and promote greater independence in daily activities.

The study included two groups of variables: sociodemographic variables (age and sex) and clinical variables related to functional performance and reason for admission.

Functional status was assessed using three indicators of recovery: the MBI for independence in ADL (Shah et al., 1989), Daniels and Worthingham’s Muscle Testing (DWMT) for muscle strength (MS) (Peláez-Vélez et al., 2023; Tolmacheva et al., 2017) and the Functional Ambulation Category (FAC) for walking ability (Mehrholz et al., 2007). For each measure, information was collected regarding pre-hospitalization status, admission, and discharge.

Causes of admission were classified into seven groups: acquired brain injury (ABI), hip fracture, other orthopedic or traumatological conditions, respiratory infection, multifactorial functional decline, falls, and other causes. The ABI category included ischemic and hemorrhagic stroke, traumatic brain injury, and brain tumors (Juárez-Belaúnde et al., 2024), while elective hip and knee arthroplasties were categorized within orthopedic conditions (Baztán Cortés et al., 2020). The “other causes” group comprised conditions such as urinary tract infection, heart failure, cardiac surgery, epilepsy, cancer, and general clinical deterioration.

Data were collected retrospectively from medical records after hospital discharge and compiled by a single researcher to ensure consistency. Assessments were performed by trained professionals following standardized procedures, and evaluations were postponed in clinically unstable patients until stabilization. In cases of readmission within two months, only the first admission was included, and in cases where multiple concurrent diagnoses were present, the admission category was assigned according to the condition considered by the multidisciplinary clinical team to be the primary cause of the functional impairment leading to admission to the Functional Recovery Unit. This decision was based on the diagnosis judged to have the greatest impact on the patient’s functional status and rehabilitation needs, following the routine clinical assessment performed at admission.

Patient profiles were identified through an unsupervised analysis based on the temporal evolution of the MBI. To better characterize clinical trajectories, clustering was not performed directly on the original MBI measurements, but on a transformed representation consisting of the baseline condition and subsequent changes between time points. Specifically, the variables included the initial pre-event level together with the changes from the pre-event stage to admission (Functional Decline) and from admission to discharge (Functional Recovery). This representation separates initial status from temporal evolution and facilitates the identification of clinically meaningful progression patterns.

Clustering of the functional trajectories was performed using a k-means algorithm (Lloyd, 1982) applied to standardized variables, where the selection of the appropriate number of groups (four) was based on the silhouette score (Rousseeuw, 1987), which balances cluster compactness and separation. The procedure was repeated using different random seeds to yield highly consistent solutions and ensure that the results did not depend on initial centroid placement. Compared with alternative criteria such as Davies–Bouldin, Calinski–Harabasz, or Dunn indices (Ikotun et al., 2025), silhouette analysis provides an interpretable and robust measure of clustering quality, particularly suitable for complex multidimensional datasets exhibiting partially overlapping structures (G. Kim et al., 2025; Shin et al., 2023).

The robustness of the resulting segmentation was initially evaluated by measuring the Jaccard similarity among the alternative partitions obtained from applying the k-means algorithm to 100 bootstrapped samples with replacement of the dataset (Hennig, 2007). Stability was quantified using the mean Jaccard similarity coefficient, where values above 0.85 indicate highly stable clusters. The study yielded > 0.92 Jaccard similarity index values, indicating that the partition is stable and reproducible.

To further assess the consistency of the identified segmentation, hierarchical clustering using Ward’s linkage on standardized variables was also applied (Ward, 1963). The resulting dendrogram showed a clear partition at the selected number of clusters (four) for the k-means, and the contingency table analysis together with the values of the Adjusted Rand Index (ARI), the Normalized Mutual Information (NMI) and the Adjusted Mutual Informations (AMI), demonstrated substantial agreement between hierarchical and k-means assignments. In parallel, a density model using Gaussian Mixture Model (GMM) (Dempster et al., 1977) was developed to explore the probabilistic structure underlying the data. The GMM algorithm selected the number of mixture models and covariate structure according to the Bayesian Information Criterion (BIC) which balances the model’s fit against its complexity. The resulting model presented a larger number of mixture components (G = 9), to be interpreted as local refinements rather than distinct clinical subgroups since the posterior probabilities and component overlap revealed substantial continuity in the data distribution. Nevertheless, hierarchical clustering applied to the GMM component centroids recovered a higher-level organization consistent with the four-cluster solution obtained with k-means and hierarchical clustering.

Once the clusters had been established, the patient distribution was visualized in the original three-dimensional MBI space using scatterplot representations in which each patient was colored according to her/his cluster membership. These visualizations showed a predominantly continuous distribution with identifiable macro-groups corresponding to the cluster partition. In addition, the average temporal MBI trajectories associated with each cluster were computed to characterize the typical functional evolution patterns represented by each patient group.

Next, the previously identified clusters were used to study the admission-to-discharge evolution of the MS and FAC variables categorized by group. For each cluster, joint (admission-and-discharge) as well as marginal distributions were graphically represented, and the corresponding temporal evolution patterns were analyzed separately. This approach enabled the comparison of functional progression in MS and FAC variables across patient profiles.

Finally, the main demographic and clinical characteristics were also comparatively studied for each cluster. To assess the significance of these differences, Kruskal–Wallis tests were performed for continuous and ordinal variables and Chi-squared tests were applied to binary and categorical variables.

All the analyses were performed using the R statistical package (version 4.4.3).

## 3. Results

### 3.1. Baseline Characteristics

The study sample included 957 patients, with women accounting for 54.96% of the population (n = 526). The mean age was 82.41 years, and the majority of participants were between 76 and 90 years old (70.95%), followed by individuals aged 60–75 years (17.86%) and those over 91 years (11.18%). The baseline demographic and clinical characteristics of the sample, categorized by sex, are summarized in Table 1.

### 3.2. Identification and Validation of the Clustering Structure

To characterize patient trajectories, we represented functional status using three clinically meaningful dimensions: baseline functional status (MBI_PRE), functional decline before admission (MBI_ADM − MBI_PRE), and functional decline during inpatient rehabilitation (MBI_DIS − MBI_ADM). Although these variables are mathematically derived from the original MBI measurements and therefore contain the same underlying information, this representation was selected because it aligns with the clinical stages of the rehabilitation process and facilitates the interpretation of functional trajectories. The aim was to express the data in a way that reflects the temporal sequence of functional decline and recovery.

**Figure 2:**
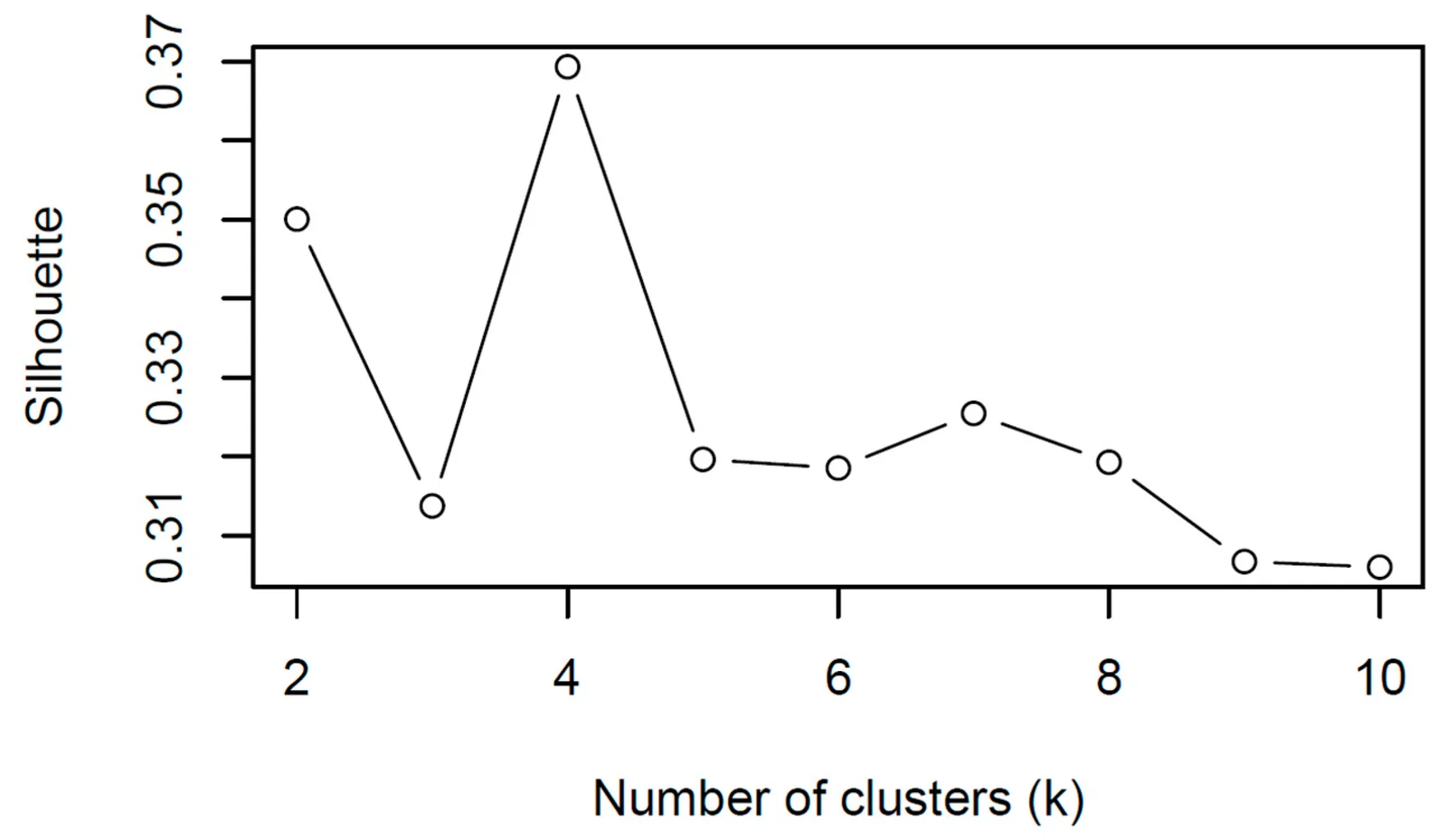
Silhouette analysis for optimal cluster selection. Circles denote the silhouette values obtained for each number of clusters (k); connecting lines are included only to guide the eye and illustrate the trend across consecutive values of k.

Clustering was first performed applying the k-means algorithm on the derived variables. The number of clusters was selected to be k = 4, according to the silhouette score, which indicated a clear improvement at this value, strongly diminishing the score beyond it, reaching a value of approximately 0.37 (Figure 2). This choice of k balances cohesion and separation while avoiding over-segmentation.

To assess the robustness of this partition, a Bootstrap-based stability analysis (with B = 100 bootstrap samples) was performed yielding very high Jaccard index values for all clusters (>0.92), indicating that the k-means partition is highly reproducible under resampling.

**Figure 3:**
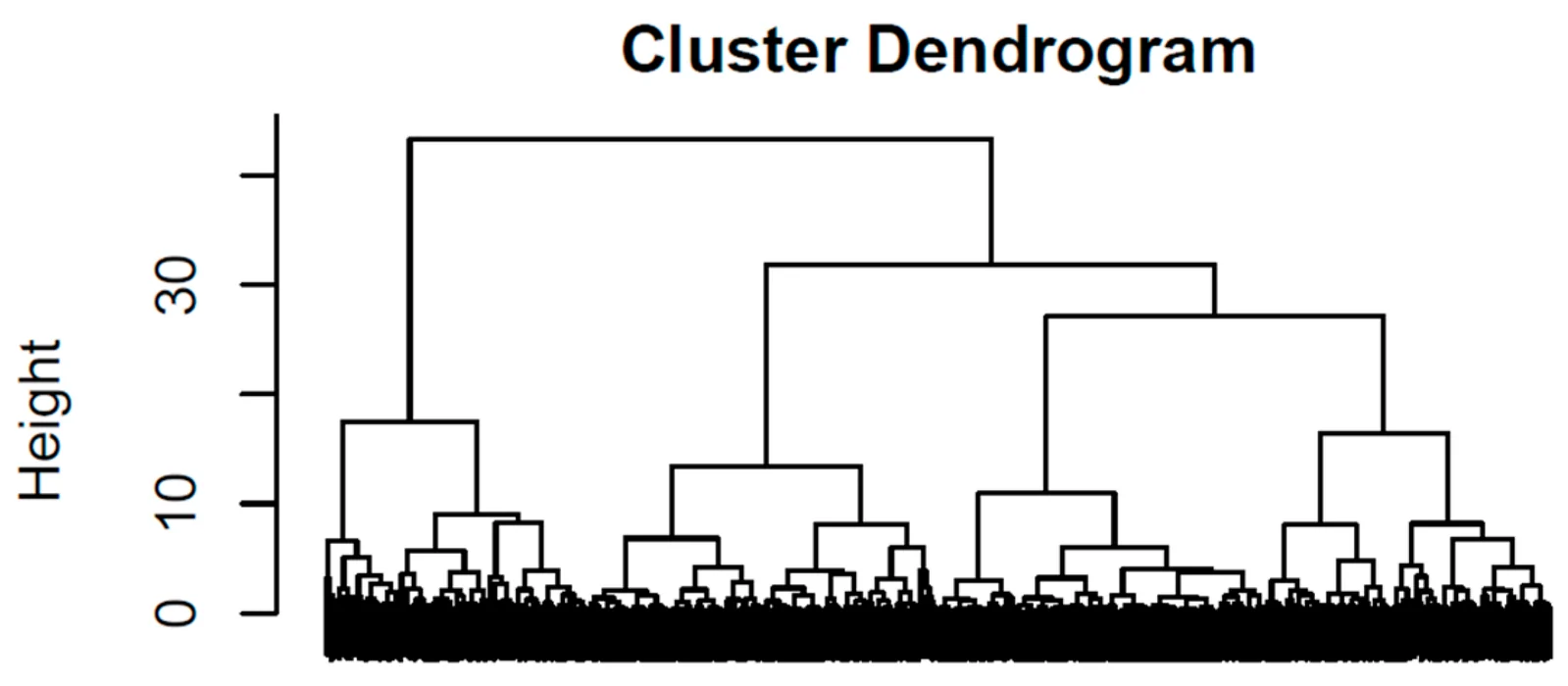
Dendrogram obtained from hierarchical clustering using Ward’s method.

To further evaluate the robustness of the segmentation, hierarchical clustering (using Ward’s method on standardized variables) was also applied. In the resulting dendrogram (Figure 3), the vertical axis corresponds to the linkage distance (i.e., the increase in within-cluster variance when two clusters are merged). A cut at k = 4 can be very well defined, since a substantial decrease in the linkage distance is required to obtain more than four clusters, indicating that the four-cluster solution captures the primary structure of the data.

The agreement between k-means and hierarchical clustering was evaluated through a contingency table (Table 2), showing strong correspondence for three of the four groups, while the remaining group displayed partial mixing. This pattern suggests that most clusters are well-defined, with one group capturing transitional cases located between more distinct regions of the data space. These results were also supported by computing several validity metrics, yielding an Adjusted Rand Index (ARI) of 0.64, a Normalized Mutual Information (NMI) of 0.65, and an Adjusted Mutual Information (AMI) of 0.65. These values indicate substantial agreement between the independent clustering approaches, confirming the stability of the four-group macro-structure.

We further explored the data structure building a density estimator with GMMs, which provided a solution with nine components according to the Bayesian Information Criterion (BIC). However, inspection of posterior probabilities and component means revealed substantial overlap between components and smooth variation along the three dimensional space of the studied variables (baseline level and changes). This indicates that the additional components provided by the GMM (when compared with the four groups given by the clustering scheme) reflect local refinements required to fit the smoothness of the distribution, rather than distinct clinical subgroups. Consistently, applying hierarchical clustering to the GMM component centroids yields a clear partition into four higher-level groups, aligned with the k-means and hierarchical clustering results.

Overall, although the silhouette score (0.37) suggests moderate separation, the combined evidence from the k-means silhouette scores, the Jaccard index values between bootstrapped-based partitions, the agreement with the hierarchical clustering results, and the GMM analysis supports a representation of the data as a partially continuous structure with four robust and interpretable macro-level groups, within which finer-grained variability is present.

### 3.3. Three-Dimensional Visualization of the Dataset Showing the Four-Cluster Structure

**Figure 4:**
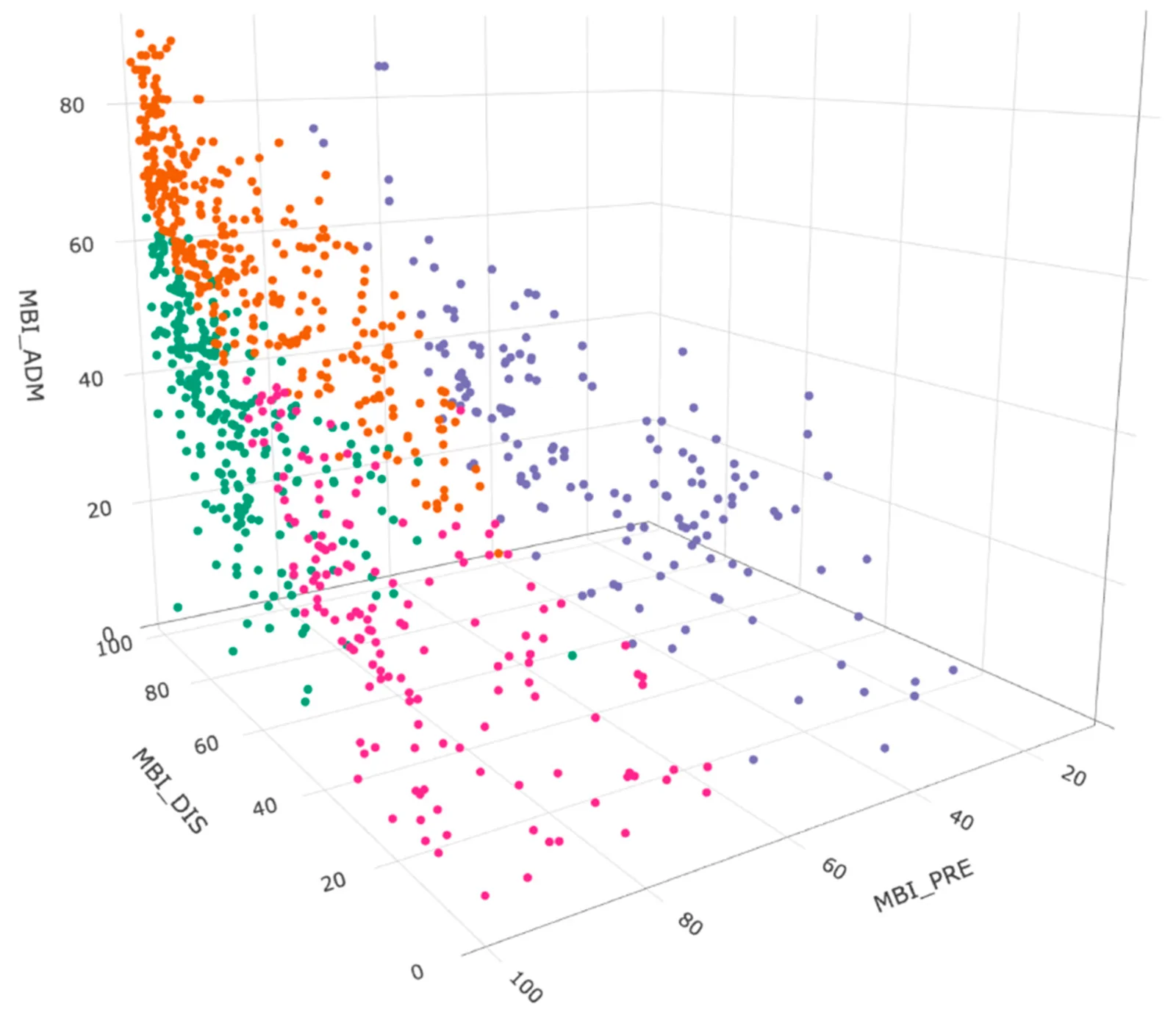
Three-dimensional representation of the cluster distribution based on MBI scores. Each dot represents an individual participant, and different colors indicate membership in different clusters.

The dataset showing the four-cluster segmentation was then visualized in the original variable space (MBI_PRE, MBI_ADM, MBI_DIS). The data points form a continuous, pyramid-like structure, reflecting gradual transitions in clinical trajectories. Within this continuum, the four clusters can still be discerned as coherent regions, supporting their interpretation as meaningful macro-groups embedded in an underlying continuous distribution. The graphical representation (Figure 4) shows the distribution across the rehabilitation process and reveals the presence of four visually differentiated patient groups, identified by the colors orange, green, pink, and purple.

The orange cluster starts with a baseline level close to independence regarding autonomy, and demonstrates the highest functional status at admission and the greatest recovery at discharge.

The green and pink clusters also start from similar baseline levels close to independence but they occupy intermediate positions regarding pre-hospitalization autonomy, both showing markedly different recovery trajectories. While the green group gathers low-to-moderate dependency admission scores, allowing a very good recovery at discharge, the pink group exhibits the lowest hospitalization scores and the poorest recovery at discharge, remaining within severe dependency levels.

Finally, the purple cluster is mainly composed of patients with the lowest baseline functional status and discharge scores consistent with moderate dependency, showing limited recovery compared with admission values.

Overall, the distribution pattern supports the existence of distinct functional recovery profiles within the sample and reinforces the usefulness of clustering techniques for patient stratification in rehabilitation settings.

### 3.4. Cluster-Based Identification of Functional Recovery Profiles

**Figure 5:**
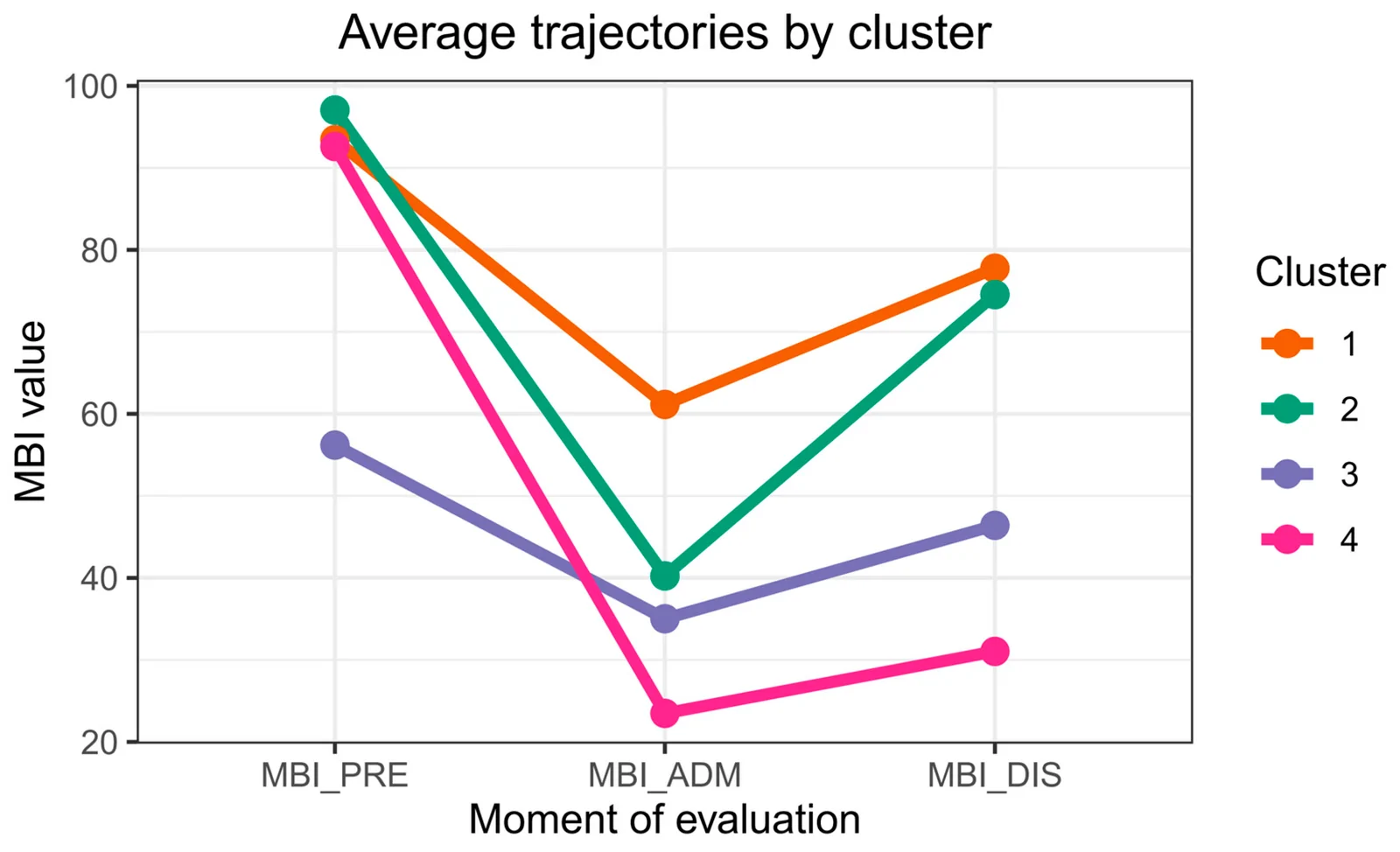
Average trajectories by cluster according to the Modified Barthel Index evolution.

The time evolution associated with each group was summarized by plotting the mean trajectories for each cluster across the three time points (Figure 5). These trajectories, (displayed using the same color code employed in Figure 4) highlight distinct patterns of decline and recovery associated with each group, providing an interpretable summary of the heterogeneous clinical dynamics captured by the clustering.

Four functional phenotypes were identified, reflecting different recovery trajectories across the rehabilitation process:

1. Group 1 (orange): Functional resilience. Patients in this group presented good baseline functional status and lower impairment at admission, recovering to levels close to full autonomy (approximately 80 points, supervised independence).
2. Group 2 (green): High recovery. This group included patients with high pre-hospitalization independence, moderate acute functional decline, and strong recovery capacity, reaching approximately 75 points at discharge, consistent with mild dependency.
3. Group 3 (purple): Chronic impairment. This profile included patients with moderate baseline dependency, smaller functional fluctuations during hospitalization, and modest recovery trajectories, reaching approximately 45 points at discharge, corresponding to moderate dependency.
4. Group 4 (pink): Severe damage. These patients showed good pre-admission functional status but experienced a marked functional decline at admission (reaching around 20 points), with limited recovery during hospitalization, reaching approximately 30 points at discharge, consistent with severe dependency.

The identified recovery trajectories improve the understanding of the diversity of functional evolution observed during geriatric rehabilitation.

### 3.5. Functional Characteristics Within Each Group

**Figure 6:**
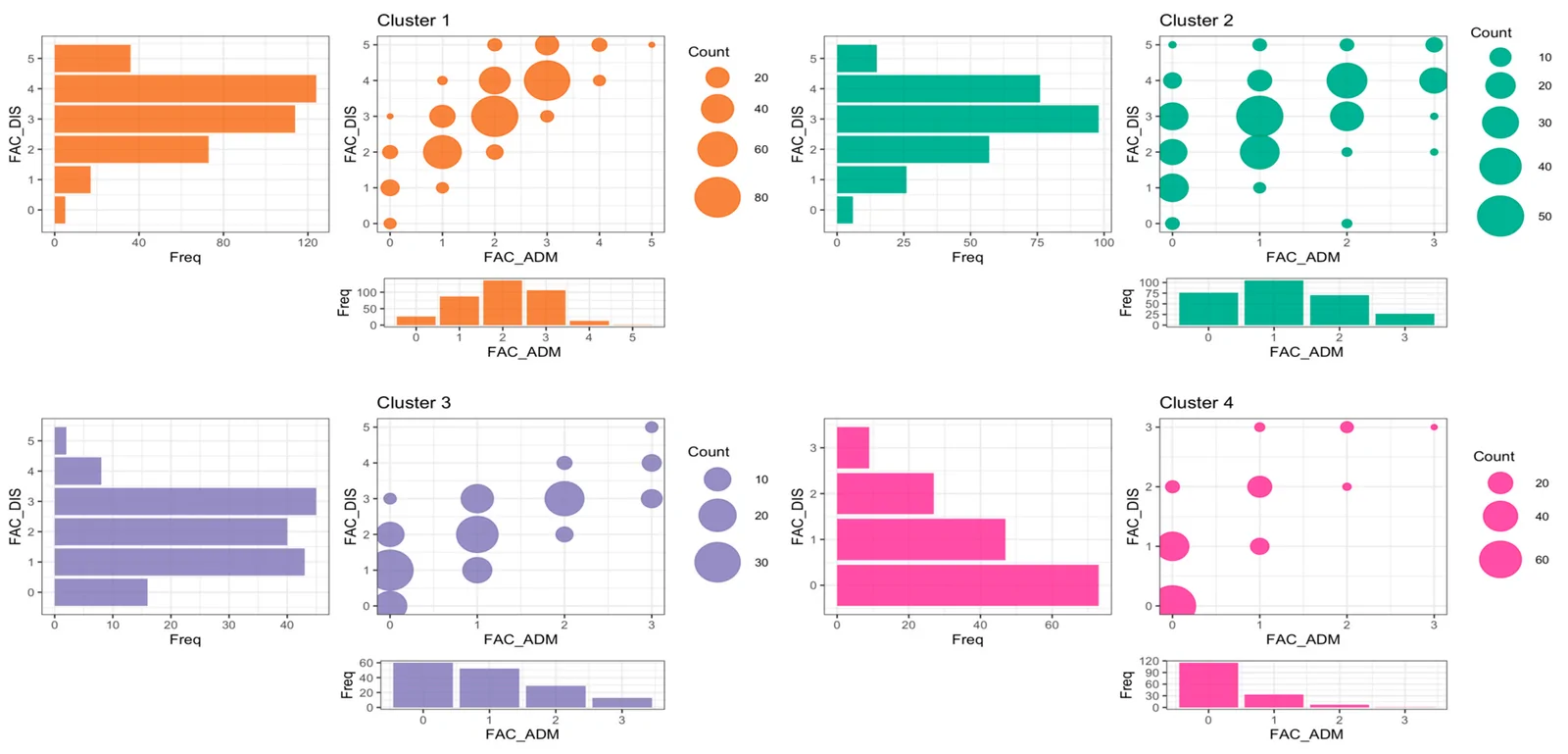
Distribution and evolution of FAC scores across the four identified clusters. Abbreviations. Freq: Frequency.

**Figure 7:**
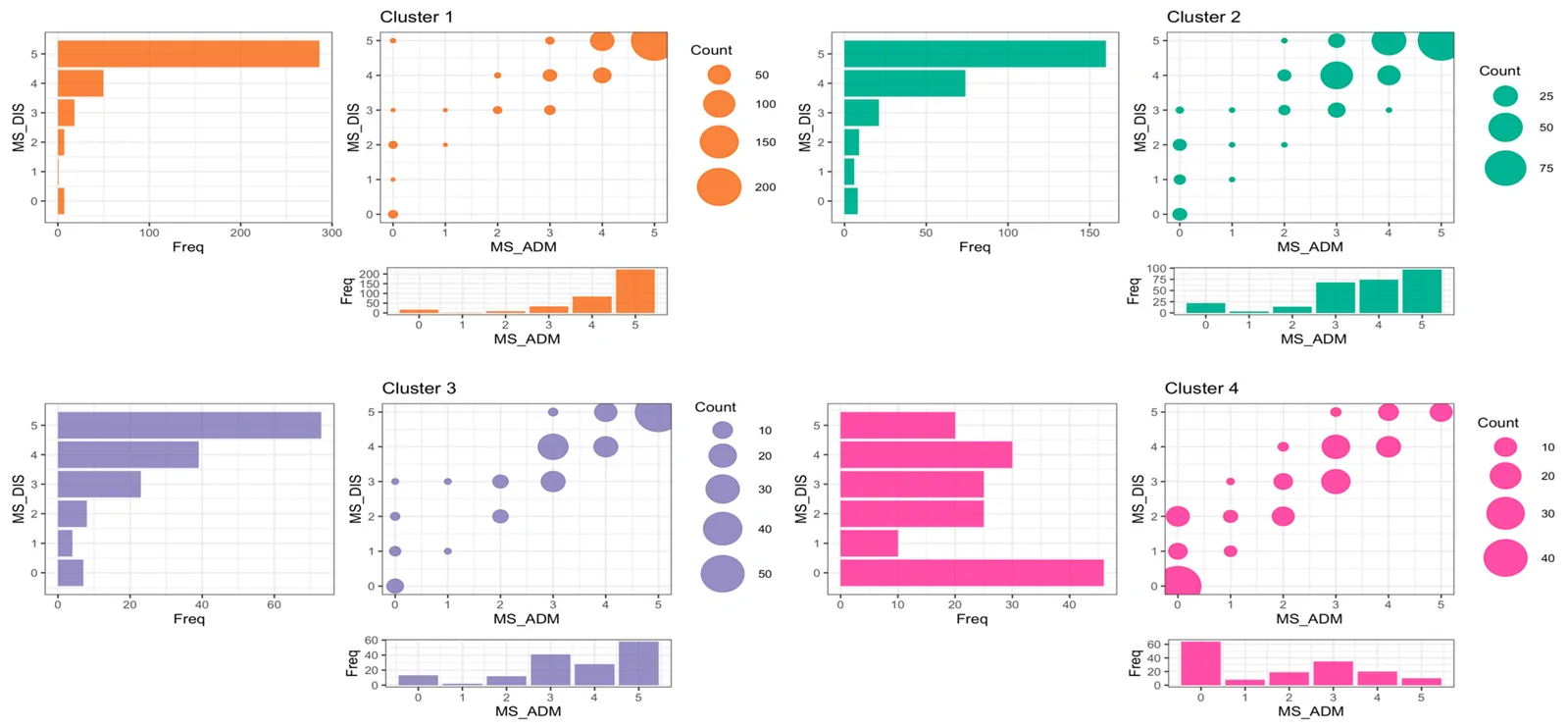
Distribution and evolution of MS scores across the four identified clusters.

Finally, the functional evolution within each one of the four groups was further analyzed by comparing changes in ambulation capacity, assessed using the FAC (Figure 6), and muscle strength, measured with the DWMT (Figure 7), from admission to hospital discharge.

Groups 1 (orange) and 2 (green) showed the most favorable gait recovery trajectories. However, Group 1 displayed a more resilient functional profile, maintaining the highest scores both at admission and discharge, and being the only cluster in which patients achieved the highest FAC levels (scores 4 and 5) from admission onwards. The observed differences in FAC at admission across the identified recovery trajectories provide additional clinical characterization of the functional patterns identified by the clustering analysis. These findings are consistent with the conceptual relationship between FAC and the MBI, as the latter includes several mobility-related items, including walking ability, transfers, and stair use. Therefore, FAC should be considered a complementary measure that helps characterize the identified recovery trajectories, rather than independent evidence supporting the validity of the clustering solution.

Group 3 (purple) also demonstrated a relevant improvement in ambulation capacity, although starting from a more compromised functional condition at admission, which was likewise reflected in discharge scores. In contrast, Group 4 (pink) showed minimal changes throughout the rehabilitation process, remaining relatively stable between admission and discharge and representing the profile with the greatest functional limitations. In fact, this group did not achieve FAC levels higher than 3 at either admission or discharge.

Regarding MS, differences between Groups 1 and 2 were less pronounced, with both clusters achieving similar recovery levels. In contrast, Group 3 showed a more favorable evolution than Group 4, reaching muscle strength outcomes comparable to those observed in the higher-performing clusters and achieving the highest muscle strength levels at discharge. Conversely, Group 4 once again demonstrated the poorest recovery trajectory, remaining consistently at the lowest muscle strength levels from admission to discharge. To complement the graphical representation of FAC and MS evolution, we implemented formal multivariate statistical comparisons of the admission–discharge distributions across the identified recovery profiles. Specifically, we applied a PERMANOVA analysis using the paired admission and discharge values as a two-dimensional response. The analysis showed a highly significant association between recovery profile and the joint distribution of FAC (pseudo-F = 175.98, R^2^ = 0.356, *p* < 0.001) and MS (pseudo-F = 119.6, R^2^ = 0.274, *p* < 0.001). Given that these variables are available only at admission and discharge, such joint admission–discharge distribution provides an appropriate representation of their clinical evolution across the groups.

To facilitate the clinical interpretation of the identified recovery trajectories, the main demographic and clinical characteristics of each cluster are summarized in Table 3. The table includes age, sex distribution, length of stay, cognitive status, social support, the most frequent reason for admission, pre-event functional status (MBI_PRE), functional status at admission (MBI_ADM) and discharge (MBI_DIS), the corresponding MBI change scores, FAC at admission and discharge, and MS at admission and discharge. To assess the significance of these differences, Kruskal–Wallis tests were performed for continuous and ordinal variables and Chi-squared tests were applied to binary and categorical variables. Together, these variables provide a comprehensive clinical characterization of the four identified recovery trajectories and facilitate the interpretation of their functional evolution.

For instance, Group 3 (Chronic Impairment) was characterized by the highest median age (86.0) and significant cognitive disorder (CD) (median CD = 2.0), while Group 4 (Severe Damage) showed the longest length of stay (median = 45 days) and the lowest discharge scores. Chi-squared tests also showed a significant association with the primary reason for admission (*p* < 0.001, V = 0.301), with Group 4 being predominantly composed of Acquired Brain Injury cases (85.3%).

## 4. Discussion

The present study aimed to identify functional recovery profiles using ML techniques in order to individualize rehabilitation interventions in a heterogeneous geriatric population. Stratification through clustering techniques provides a robust quantitative framework that complements the clinical reasoning of occupational therapists. By identifying typical recovery profiles, clinicians may gain a better understanding of the heterogeneity of functional recovery among older adults undergoing geriatric rehabilitation. It is important to emphasize that these profiles represent retrospective recovery trajectories. Whether they can serve as a foundation for developing future predictive models to support individualized intervention planning based on admission characteristics remains to be determined.

However, the high methodological complexity of many of these approaches, together with the limited interpretability of certain algorithms, hinders their integration into routine clinical practice. Furthermore, previous systematic reviews have identified important methodological limitations in this type of research, reducing the robustness and generalizability of the reported findings (Veerbeek et al., 2011).

Providing a representation more closely to real-world geriatric rehabilitation settings, one of the main strengths of this study lies in the analysis of a large and clinically diverse cohort composed of older inpatients with multiple etiologies associated with functional decline. In this context, the AIRCOT project aims to provide a realistic and clinically applicable solution tailored to the needs of geriatric patients.

Regarding the findings of the present study, the identification of four functional recovery profiles is consistent with the results previously reported by Shin et al., who also identified four main clusters in patients with hemorrhagic stroke and five clusters in those with ischemic stroke (Shin et al., 2023). This approach enables the identification of subgroups of patients with similar characteristics, which may facilitate the personalization of rehabilitation interventions, as also suggested in previous studies (Majcen Rosker & Rosker, 2026; Twumasi et al., 2025). In turn, Armstrong and colleagues identified seven distinct patient groups within geriatric rehabilitation populations (Armstrong et al., 2012); however, their variables were analyzed from a preventive perspective in patients’ home environments prior to the onset of the functional decline that later led to hospitalization. Consequently, those groups are more closely related to prevention profiles rather than intervention-oriented profiles, as in the present study. In contrast, Albu and colleagues identified three distinct groups in their study (Albu et al., 2024), which appears to be more closely aligned with our findings, as their study population was similar to ours.

Regarding the use of the MBI as the main determinant of cluster formation, this approach is also supported by previous studies. Shin and colleagues had already employed the MBI as a functional measure to identify different recovery profiles (Shin et al., 2023), as previously reported by Lin and colleagues in their study applying ML techniques (Lin et al., 2018). In contrast, other authors have chosen to use the Functional Independence Measure (FIM), such as Miyazaki (Miyazaki et al., 2023) and the previously mentioned study by Albu (Albu et al., 2024). Nevertheless, all these studies agree on the importance of assessing independence in ADLs when defining rehabilitation profiles in geriatric populations. In particular, the MBI has gained increasing relevance in recent research, emerging as a strong predictor not only of functional recovery but also of mortality (Jaramillo-Hidalgo et al., 2026), which supports its selection as the primary measure in the present study.

Recovery of ambulation followed the expected trajectories across the four functional groups analyzed, consistent with the FAC findings previously reported in Shin’s study (Shin et al., 2023). Regarding MS evolution, the behavior of Group 3 (purple) was particularly noteworthy, as it showed muscle recovery levels close to those observed in the higher-performing functional groups despite maintaining greater limitations in ambulation capacity. This finding may suggest a partial dissociation between muscle strength recovery and gait-related functional recovery in certain clinical profiles. A similar pattern was observed in Shin’s study (Shin et al., 2023) where the Fugl–Meyer Test also demonstrated a more favorable evolution than ambulation outcomes. Although both scales provide comparable information regarding motor recovery, the use of different assessment tools should be considered when interpreting these comparisons. The current literature shows no clear consensus regarding the optimal instrument for muscle strength evaluation. In the present study, the DWMT was selected because it is a widely used rehabilitation tool due to its ease of clinical application, low cost, and adequate inter-rater reliability for muscle strength assessment (Bohannon, 2018; Naqvi et al., 2025).

An important aspect of the proposed clustering approach is that it incorporates temporal information on functional status by representing patients according to their baseline condition and subsequent functional changes throughout the rehabilitation process. Consequently, the identified clusters should be interpreted as retrospective recovery trajectories, providing a clinically meaningful description of the different patterns of functional evolution observed in the study population. Rather than serving as predictive models, these recovery profiles improve the understanding of the heterogeneity of rehabilitation outcomes and may provide a conceptual framework for the future development of supervised predictive models based exclusively on admission characteristics. Such predictive approaches, however, are beyond the scope of the present study.

In general, the identification of distinct retrospective recovery subgroups improves the understanding of heterogeneity in rehabilitation outcomes and may serve as a foundation for future predictive approaches aimed at supporting more individualized intervention protocols. In this context, OT plays a key role in interpreting findings such as those observed in Group 3, where improvements in muscle strength were not directly associated with proportional gains in functional performance. The occupational therapist’s perspective may help identify whether environmental, cognitive, or contextual factors are mediating this discrepancy, supporting the usefulness of clustering models for the early detection of “low functional resilience” profiles that require targeted interventions focused on occupational performance. For instance, a patient belonging to Group 4 in our study (severe damage), who presented a pre-admission score of 90 points but declined to 20 points at admission with limited subsequent recovery, represents a phenotype of low functional resilience that may require early intensive rehabilitation protocols or further assessment of underlying medical factors limiting functional progress. In this way, statistical findings may be translated into clinically meaningful precision rehabilitation strategies.

Among the main limitations of the present study are its retrospective design and the fact that the analyzed sample was obtained from a single specialized geriatric rehabilitation unit, which may limit the generalizability of the findings. In addition, the specific admission criteria applied in this unit, including a pre-admission Modified Barthel Index (MBI) > 40 and limited cognitive impairment, resulted in a clinically homogeneous study population with rehabilitation potential. While these criteria reflect routine clinical practice within our Functional Recovery Unit and were intended to ensure an appropriate cohort for the analysis of functional recovery trajectories, they also defined a specific rehabilitation population, which may limit the external validity and generalizability of the findings to broader geriatric populations. Furthermore, local admission policies, rehabilitation protocols, and characteristics of the Spanish healthcare system may have influenced the observed recovery trajectories. Consequently, the identified recovery trajectories should be interpreted within the context of specialized post-acute geriatric rehabilitation, and caution should be exercised when extrapolating these findings to other institutions, countries, or healthcare systems. Likewise, the absence of certain environmental and contextual variables, together with the methodological sensitivity inherent to clustering algorithms, may have influenced both the configuration of the identified recovery trajectories and their clinical interpretation. Moreover, the identified clusters should be understood as meaningful macro-patterns within a fundamentally continuous distribution rather than as sharply separated natural subtypes. Although the recovery trajectories were internally stable, they have not yet been externally validated in other healthcare settings, and their generalizability should be confirmed through future prospective multicenter studies.

The recovery trajectories identified in this study provide a structured description of the heterogeneity of functional evolution observed during post-acute geriatric rehabilitation. Although these trajectories may contribute to a better understanding of patient recovery patterns and generate hypotheses for future research, the present analysis does not demonstrate that assigning patients to these trajectories improves clinical decision-making or rehabilitation outcomes. Future prospective and multicenter studies are needed to evaluate whether these recovery trajectories can support clinically actionable stratification strategies and guide individualized rehabilitation interventions based on patients’ baseline characteristics.

In addition, progress toward techniques compatible with their integration into routine clinical practice will also be necessary. In this regard, the continuation of the AIRCOT project could contribute to the development of functional recovery predictive models combined with the clustering techniques used in this study, generating more precise stratification and prediction systems tailored to geriatric rehabilitation, as suggested by Elbattah’s study (Elbattah & Molloy, 2017).

## 5. Conclusions

In conclusion, unsupervised machine learning clustering techniques show considerable potential for identifying functional recovery profiles in heterogeneous geriatric rehabilitation populations. The application of these approaches may improve the understanding of functional recovery patterns and support the future development of predictive tools for clinical stratification and more individualized OT interventions.

## Figures and Tables

**Table 1:** Baseline characteristics of the study population.

	Overall	Female	Male
N	957	526	431
Age (mean (SD))	82.41 (6.7)	82.94 (6.7)	81.74 (6.9)
Reason for admission (N (%))			
ABI	422 (44.2)	209	213
HF	180 (18.8)	125	55
OP	137 (14.3)	97	40
RTI	99 (10.3)	36	63
MC	22 (2.3)	11	11
AF	51 (5.3)	31	20
Others	46 (4.8)	17	29
Time from functional decline to admission (days) (mean (SD))	18.75 (17.5)	17.96 (16.9)	19.71(18.2)
MBI (mean (SD)			
PRE	88.36 (16.7)	87.47 (17.0)	89.44 (16.3)
ADM	44.73 (19.3)	45.45 (19.6)	43.86 (18.8)
DIS	64.17 (23.2)	64.16 (23.4)	64.17 (22.8)
MS (mean (SD))			
ADM	3.58 (1.6)	3.68 (1.7)	3.44 (1.6)
DIS	4.02 (1.5)	4.03 (1.5)	4 (1.4)
FAC (mean (SD))			
ADM	1.32 (1.1)	1.4 (1.1)	1.21 (1.1)
DIS	2.53 (1.4)	2.55 (1.38)	2.5 (1.4)

For all quantitative variables, the mean and the standard deviation are reported. For qualitative variables the total values (N) and their percentages are shown. The dataset contained no missing values across all clinical and sociodemographic variables analyzed in this study. Abbreviations. SD: Standard Deviation; ABI: Acquired Brain Injury; HF: Hip Fracture; OP: Orthopedic Procedure; RTI: Respiratory Tract Infection; MC: Multifactorial Causality; AF: Accidental Fall; MBI: Modified Barthel Index; PRE: Previous Situation; ADM: Patient Admission; DIS: Patient Discharge; MS: Muscle Strength; FAC: Functional Ambulation Category.

**Table 2:** Contingency table comparing cluster assignments obtained by k-means (rows) and hierarchical clustering (columns, rearranged for easier visualization).

Clusters	3	2	4	1
1	252	6	60	51
2	3	263	2	10
3	0	0	143	11
4	0	4	0	152

**Table 3:** Clinical characteristics of the four recovery groups.

Variable	Group 1: Functional Resilience (n = 369)	Group 2: High Recovery (n = 278)	Group 3: Chronic Impairment (n = 154)	Group 4: Severe Damage (n = 156)	Test	*p*-Value	Effect Size	Int.
AGE	82.0 [76.0–87.0]	83.0 [77.0–86.0]	86.0 [80.0–90.0]	83.0 [77.0–89.0]	K-W	<0.001	ε^2^ = 0.030	S
STAY	21.0 [14.0–30.0]	34.0 [25.0–45.8]	27.0 [16.0–34.0]	45.0 [25.8–63.2]	K-W	<0.001	ε^2^ = 0.189	M
TIME FROM FUNCTIONAL DECLINE TO ADMISSION	10.0 [6.0–20.0]	13.5 [7.0–20.8]	16.0 [10.0–29.8]	20.0 [14.0–30.0]	K-W	<0.001	ε^2^ = 0.080	M
MBI_PRE	98.0 [87.0–100.0]	100.0 [95.0–100.0]	60.0 [47.2–66.0]	98.0 [85.0–100.0]	K-W	<0.001	ε^2^ = 0.456	L
MBI_ADM	62.0 [52.0–70.0]	40.0 [31.2–50.0]	34.0 [24.2–44.0]	23.0 [15.0–31.0]	K-W	<0.001	ε^2^ = 0.550	L
MBI_DIS	80.0 [69.0–90.0]	75.0 [66.0–85.0]	48.0 [35.0–58.0]	30.0 [21.0–42.0]	K-W	<0.001	ε^2^ = 0.555	L
FUN_DECLINE	−33.0 [−39.0–25.0]	−55.0 [−64.0–−48.0]	−22.0 [−30.0–−13.0]	−69.0 [−77.0–−59.0]	K-W	<0.001	ε^2^ = 0.726	L
FUN_RECOV	17.0 [12.0–22.0]	33.0 [28.2–39.0]	10.0 [4.0–17.0]	6.0 [2.0–13.0]	K-W	<0.001	ε^2^ = 0.636	L
CD	0.0 [0.0–1.0]	0.0 [0.0–1.0]	2.0 [0.0–2.0]	2.0 [1.0–3.0]	K-W	<0.001	ε^2^ = 0.220	M
FAC_ADM	2.0 [1.0–3.0]	1.0 [0.0–2.0]	1.0 [0.0–2.0]	0.0 [0.0–1.0]	K-W	<0.001	ε^2^ = 0.309	L
FAC_DIS	3.0 [2.0–4.0]	3.0 [2.0–4.0]	2.0 [1.0–3.0]	1.0 [0.0–1.0]	K-W	<0.001	ε^2^ = 0.363	L
MS_ADM	5.0 [4.0–5.0]	4.0 [3.0–5.0]	4.0 [3.0–5.0]	2.0 [0.0–3.0]	K-W	<0.001	ε^2^ = 0.237	M
MS_DIS	5.0 [5.0–5.0]	5.0 [4.0–5.0]	4.0 [3.0–5.0]	2.0 [0.0–4.0]	K-W	<0.001	ε^2^ = 0.265	L
GENDER					C-S	0.006	V = 0.114	S
FEMALE	223 (60.4%)	133 (47.8%)	91 (59.1%)	79 (50.6%)				
MALE	146 (39.6%)	145 (52.2%)	63 (40.9%)	77 (49.4%)				
SS					C-S	<0.001	V = 0.144	S
NO	81 (22.0%)	61 (21.9%)	56 (36.4%)	54 (34.6%)				
YES	288 (78.0%)	217 (78.1%)	98 (63.6%)	102 (65.4%)				
REASON FOR ADMISSION					C-S	<0.001	V = 0.301	M
ABI	96 (26.0%)	130 (46.8%)	63 (40.9%)	133 (85.3%)				
HF	87 (23.6%)	48 (17.3%)	38 (24.7%)	7 (4.5%)				
OP	111 (30.1%)	21 (7.6%)	5 (3.2%)	0 (0.0%)				
RTI	29 (7.9%)	48 (17.3%)	17 (11.0%)	5 (3.2%)				
MC	7 (1.9%)	9 (3.2%)	4 (2.6%)	2 (1.3%)				
AF	19 (5.1%)	10 (3.6%)	16 (10.4%)	6 (3.8%)				
OTHERS	20 (5.4%)	12 (4.3%)	11 (7.1%)	3 (1.9%)				

Abbreviations. Int.: Interpretation; S: Small; M: Moderate; L: Large; K-W: Kruskal–Wallis; C-S: Chi-square; MBI: Modified Barthel Index; PRE: Previous Situation; ADM: Patient Admission; DIS: Patient Discharge; FUN: Functional; RECOV: Recovery; CD: Cognitive disorder; MS: Muscle Strength; FAC: Functional Ambulation Category; SS: Social support; ABI: Acquired Brain Injury; HF: Hip Fracture; OP: Orthopedic Procedure; RTI: Respiratory Tract Infection; MC: Multifactorial Causality; AF: Accidental Fall.

## Data Availability

The data presented in this study are available on request from the corresponding author due to privacy restrictions related to the analyzed subjects.

## Abbreviations

The following abbreviations are used in this manuscript:

ABI	Acquired Brain Injury
ADL	Activities of Daily Living
ADM	Admission/Patient Admission
AF	Accidental Falls
AI	Artificial Intelligence
BIC	Bayesian Information Criterion
CRM	Cruz Roja Mental Scale
DIS	Discharge/Patient Discharge
DWMT	Daniels and Worthingham’s Muscle Testing
FAC	Functional Ambulation Category
FIM	Functional Independence Measure
GMM	Gaussian Mixture Model
HF	Hip Fracture
MBI	Modified Barthel Index
MC	Multifactorial Causality
ML	Machine Learning
MS	Muscle Strength
OP	Orthopedic Procedures
OT	Occupational Therapy
PRE	Previous Situation/Pre-hospitalization Status
RTI	Respiratory Tract Infection
SD	Standard Deviation
TSO	Socio-Family Assessment Scale
